# Evaluation of Choroidal and Retinal Features in Patients with Primary Vasculitis—An Original Optical Coherence Tomography and Optical Coherence Tomography Angiography Study

**DOI:** 10.3390/jcm12216827

**Published:** 2023-10-29

**Authors:** Urszula Szydełko-Paśko, Joanna Przeździecka-Dołyk, Andrzej Dołyk, Artur Małyszczak, Marta Misiuk-Hojło

**Affiliations:** 1 Department of Ophthalmology, Wrocław Medical University, 50-556 Wrocław, Poland; 2 Department of Optics and Photonics, Wrocław Univeristy of Science and Technology, 50-370 Wrocław, Poland; 3 Clinic of Angiology, Systemic Hypertension and Diabetology, Wrocław Teaching Hospital, 50-556 Wrocław, Poland

**Keywords:** vasculitis, choroidal vascularity index, FAZ, circularity index, OCT, OCTA

## Abstract

Ocular manifestations have been described in the course of various types of vasculitis. However, there seems to be no routine ophthalmological examinations for patients suffering from those diseases. To ensure holistic care we aimed to investigate any retinal and choroidal abnormalities in patients suffering from primary vasculitis. The objective was to use non-invasive methods, which would not be time- and cost-consuming, yet would be helpful in routine tests. We conducted a prospective and observational study in 41 patients (78 eyes) with 5 types of primary vasculitis, including: Takayasu’s arteritis; giant cell arteritis; Buerger’s disease; granulomatosis with polyangiitis; and polyarteritis nodosa. A total of 44 healthy individuals were enrolled in the control group for comparison (88 eyes). With the use of optical coherence tomography, optical coherence tomography angiography, and MATLAB, the following parameters were assessed: choroidal thickness; vascularity index; area and perimeter of foveal avascular zone; and circularity index. The following parameters were lower in the study group compared to the control group: mean nasal and temporal CTs; mean central, temporal, and nasal CVI; and mean CI. In contrast, the results of mean central CT as well as the area and perimeter of FAZ were higher in the study group. The differences were statistically significant in the case of all parameters except for CI. Conducting routine ophthalmological examinations in patients diagnosed with vasculitis by assessment of the retina and choroid by measuring parameters like CT, CVI, area and perimeter of FAZ, and CI could be beneficial, as it may detect pathological changes before any ocular symptoms alarm the patients. CVI seems to be especially promising for choroidal evaluation, as it appears to be less influenced by various factors compared to CT.

## 1. Introduction

Vasculitis is a term for a group of rare diseases in which an inflammation of the blood vessels develops. This results in thickening of the blood vessel wall, stenosis, and eventually reduction of blood flow to various tissues and organs [1,2]. The pathological process may affect the area surrounding the lesion as well as peripheral areas and visceral organs [3]. Vasculitis can be classified according to the size of the blood vessels affected by the inflammation (large, medium, or small vessel vasculitis) or the underlying cause of the disease—primary (with no certain cause) and secondary (to other diseases or drug-induced) [3]. The pathogenesis of primary vasculitis is not fully known. A combination of genetic and autoimmune factors is usually taken into account [1,2]. In the cases of Takayasu’s arteritis (TA), giant cell arteritis (GCA), and Buerger’s disease, the involvement of various types of human leukocyte antigens (HLAs) has been documented [4,5]. Polyarteritis nodosa (PN) has been associated with immune complex deposition, while granulomatosis with polyangiitis (GPA) and eosinophilic granulomatosis with polyangiitis (EGPA) have been linked to the presence of antineutrophil cytoplasmic antibodies (ANCA) [4,6]. The diagnostic process is often challenging and involves comprehensive examinations, including laboratory and imaging tests, to rule out other systemic diseases [4]. The treatment of vasculitis typically involves the use of steroids and immunosuppressive drugs, with monoclonal antibodies also reported as beneficial [3,4].

Ocular manifestations have been documented in various types of vasculitis. Turk et al. conducted a systematic review and meta-analysis examining the involvement of the eye in systemic diseases, including GCA and GPA [7]. The prevalence of ocular disorders present in those two types of primary vasculitis was established to be 27% and 26%, respectively. Between 10% and 20% of patients diagnosed with PN develop ophthalmic or neuro-ophthalmic signs and symptoms, whereas in the case of Kawasaki disease (KD), the presence of eye disorders reaches 90% [8,9]. Our findings, described in a systematic review and meta-analysis focusing on ocular manifestations of TA, revealed that in over 74% of the analyzed cases (122 patients), ophthalmic pathologies preceded the diagnosis of TA [10]. Although the association between primary vasculitis and ocular manifestations is well-known, routine ophthalmological examinations for patients with these diseases seem to be lacking, despite the potential for systemic inflammation and ischemia to lead to severe ophthalmic complications.

To ensure holistic care we aimed to investigate any retinal and choroidal abnormalities in patients suffering from primary vasculitis. The objective was to use non-invasive methods, which would not be time- and cost-consuming, yet would be helpful in routine tests. In the case of diabetes mellitus, a systemic disease (DM), routine ophthalmological examinations have become a standard practice [11]. The goal is to detect ocular complications of the disease, even before the patients notice any symptoms. It might be worth considering such examinations for patients diagnosed with vasculitis as well. In recent years, optical coherence tomography angiography (OCTA) has gained widespread use, as an examination method. It offers increased safety compared to conventional angiography techniques, both qualitative and quantitative analysis of the retina and the choroid, as well as shorter examination time [12]. These attributes can be particularly beneficial when conducting screening or routine tests. Lately, a parameter called choroidal vascularity index (CVI), which can be calculated on the basis of OCTA results, has gained more attention. It is defined as a ratio of vascular area to the total choroidal area [13]. Evereklioglu et al. examined patients with Behcet’s disease, another type of primary vasculitis, and concluded that lower CVI values were present in patients with both active and inactive forms of ocular Behcet’s disease compared to the control group, whereas there were no differences between patients with non-ocular Behcet’s disease and the control group [14]. In our study, we decided to assess the choroid by calculating the choroidal thickness (CT) and CVI and the retina with the use of the following parameters: area and perimeter of foveal avascular zone (FAZ); and circularity index (CI). 

## 2. Materials and Methods

We conducted a prospective and observational study in 41 patients with 5 types of primary vasculitis: TA (n = 8); GCA (n = 5); Buerger’s disease (n = 11); GPA (n = 12); and PN (n = 5). A total of 78 eyes were included in the study group. A total of 4 eyes were excluded because of reduced quality of the obtained images. The diagnoses of GCA, TA, GPA, and PN were based on ACR criteria. For Buerger’s disease, Shinoaya’s criteria were used. An approval from the local Ethical Committee was granted. In addition to the diagnosis of the five selected diseases, another inclusion criterion was the presence of active disease. All patients in the study group were hospitalized due to the necessity of initiating treatment. In some cases, the diagnosis of vasculitis was newly established, while others experienced a relapse of the disease. All patients received the appropriate treatment. Ophthalmological examinations were conducted immediately before the initiation of treatment. The ages of the patients ranged from 24 to 71, with the mean value being equal to 50.9. In addition, 44 healthy individuals were enrolled in the control group for comparison (88 eyes; mean age 36.3).

The patients underwent complete ophthalmological diagnostics, in which the following examinations were performed: best-corrected visual acuity; intraocular pressure (air puff measurement); slit-lamp examination with dilated fundus examination; optical coherence tomography (OCT; Heidelberg Engineering, Heidelberg, Germany); and OCTA (RTVue XR 100 Avanti Edition, Optovue Inc., Fremont, CA, USA).

OCTA examination involved taking 6 × 6 mm scans of the retina with the fovea being in the center. The built-in software enabled the extraction of superficial and deep retinal capillary plexus images. Numerical values of the area and perimeter of FAZ as well as CI were obtained with the use of the MATLAB software (R2018a, MathsWorks, Inc., Natick, MA, USA). A low-pass filter and an active contour method were employed to determine the perimeter pixels and center of the FAZ. The following pattern was used to calculate CI: CI = 4π × (area/perimeter^2^). A more detailed description of this part of the methodology was presented by one of our co-authors in a study concerning glaucomatous macular vasculature [15]. 

The OCT examination enabled acquisition of 21 volume B-scans using the enhanced depth imaging (EDI) mode, with the scans centered on the fovea. Subsequently, images were exported for further analysis, focusing on the subfoveal choroidal area with a width of 1.0 mm. The first step involved calculating CT, which measures the vertical distance between the outer border of the retinal pigment epithelium (outside the hyperreflective line) to the border of the inner sclera (hyporeflective line). CT was determined using a previously described binarization method, which allows the conversion of a grayscale image into a binary image (comprising dark and light pixels) [16]. Following the CT calculations, the luminal area (LA) was determined, and CVI was calculated using the following formula: CVI = LA/CT. Details of this methodology are comprehensively described by Agrawal et al. [16].

For statistical analysis, MedCalc Statistical Software was used (version 22.007). The analysis was performed to determine potential differences between the study group and the control group as well as within the study group (between the subgroups consisting of patients with different types of vasculitis). The variables were assessed by the Kruskal–Wallis test and, additionally, a post-hoc analysis was conducted. The results were considered statistically significant when the value of *p* was ≤0.05.

## 3. Results

The raw data for choroidal and retinal measured variables can be found in the Supplementary Materials. Table 1 presents mean values for the following choroidal parameters: central, nasal, and temporal CTs; as well as central, nasal, and temporal CVIs. Both parameters were measured at the foveal center and at 500 μm nasally and temporally from the center. The results below are provided for the entire study group and are also presented separately for each type of vasculitis, as well as for the control group.

The mean CT in the study group equaled 341 μm, whereas in the control group, it measured 262 μm. The highest mean value was observed in PN patients, while the lowest was observed in the Buerger’s disease subgroup. While a statistically significant difference was found between the study and control groups, no significance was observed among the subgroups. Regarding central CVI, the mean value in our study group was 49.6%, compared to 64.5% in the control group, and this difference was statistically significant. The lowest mean central CVI value was found in GCA patients, while the highest value was seen in the TA subgroup. Post-hoc analysis revealed no statistically significant differences among the subgroups. 

Table 2 contains data related to mean values of retinal parameters, including the area and perimeter of FAZ, as well as CI. As in Table 1, these data are provided for the entire group, each subgroup separately, and for the control group. 

In the study group, the mean FAZ area equaled 0.34, while in the control group it measured 0.26. Similarly, the mean FAZ perimeter was higher in the study group (2.18 mm vs. 1.89 mm). GCA patients exhibited the highest values for both FAZ area and perimeter, while the PN subgroup had the lowest values. Statistically significant differences were found between the study and control groups for both FAZ area and perimeter, but no significance was observed among the subgroups. Regarding the CI, the mean values were 0.86 for the study group and 0.87 for the control group, with no significant differences between the groups.

**Figure 1:**
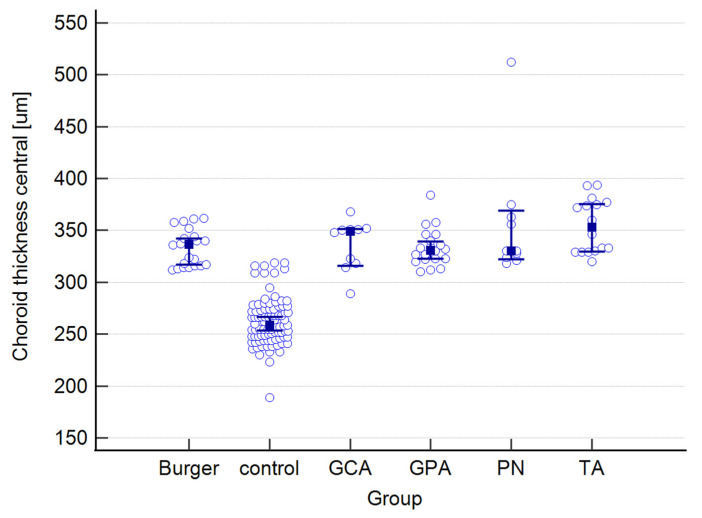
Central choroidal thickness in the study and control groups. Abbreviations: TA—Takayasu’s arteritis, GCA—giant cell arteritis, GPA—granulomatosis with polyangiitis, PN—polyarteritis nodosa.

**Figure 2:**
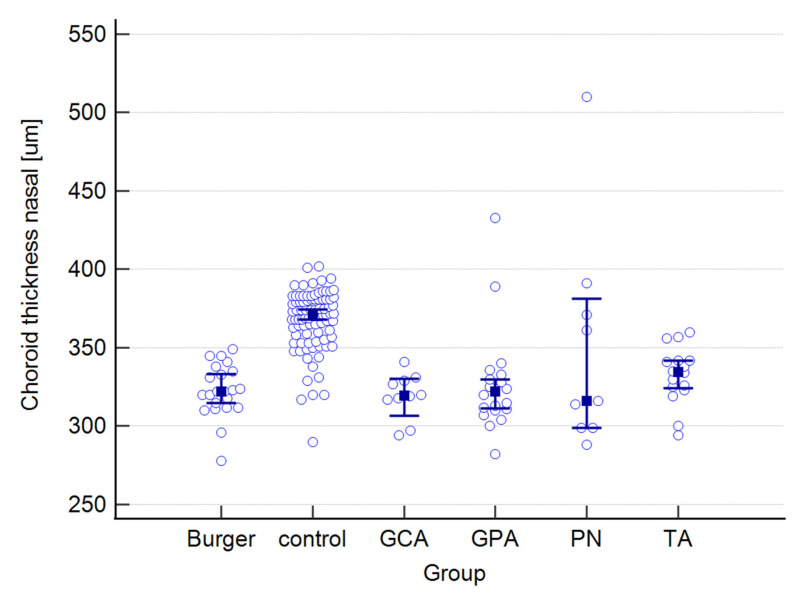
Nasal choroidal thickness in the study and control groups. Abbreviations: TA—Takayasu’s arteritis, GCA—giant cell arteritis, GPA—granulomatosis with polyangiitis, PN—polyarteritis nodosa.

**Figure 3:**
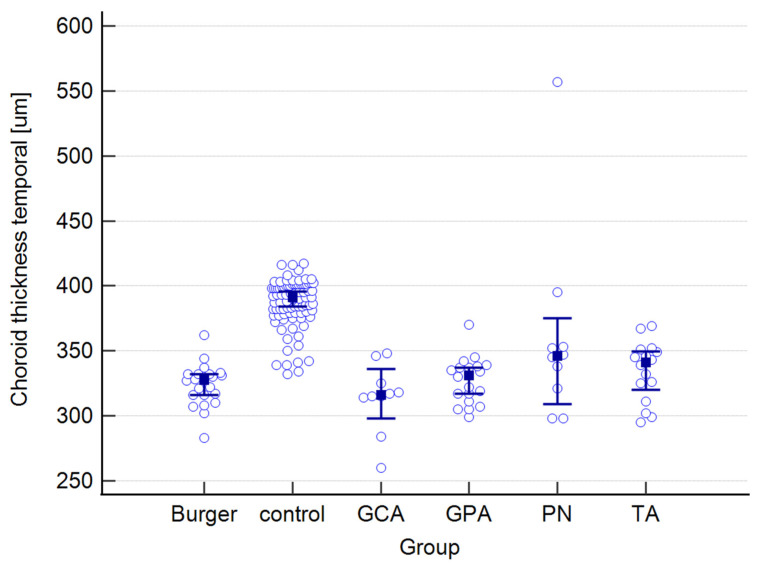
Temporal choroidal thickness in the study and control groups. Abbreviations: TA—Takayasu’s arteritis, GCA—giant cell arteritis, GPA—granulomatosis with polyangiitis, PN—polyarteritis nodosa.

**Figure 4:**
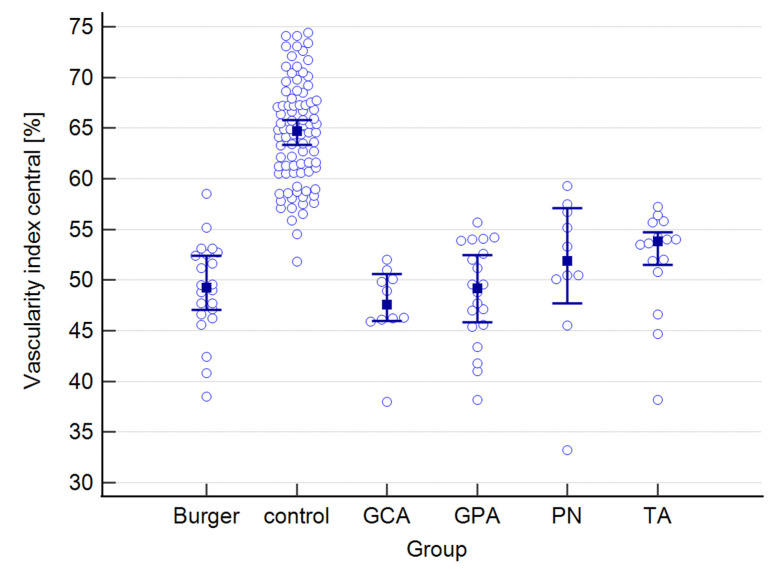
Central vascularity index in the study and control groups. Abbreviations: TA—Takayasu’s arteritis, GCA—giant cell arteritis, GPA—granulomatosis with polyangiitis, PN—polyarteritis nodosa.

**Figure 5:**
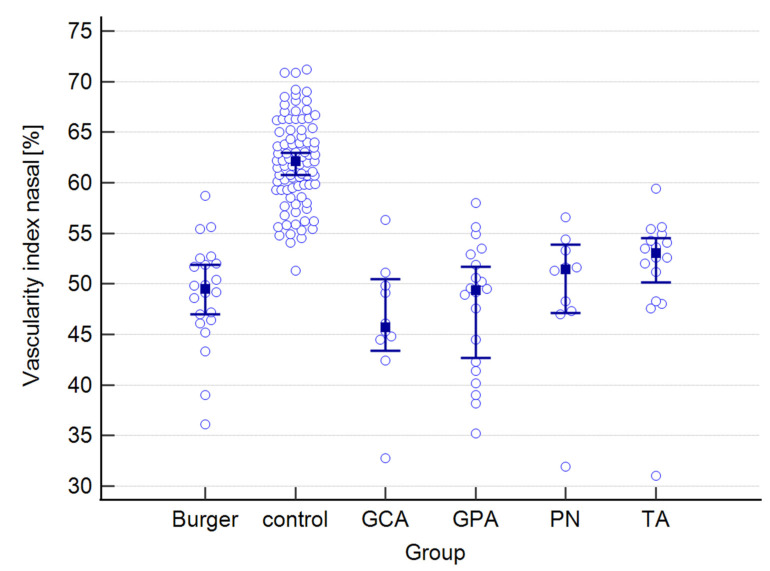
Nasal vascularity index in the study and control groups. Abbreviations: TA—Takayasu’s arteritis, GCA—giant cell arteritis, GPA—granulomatosis with polyangiitis, PN—polyarteritis nodosa.

**Figure 6:**
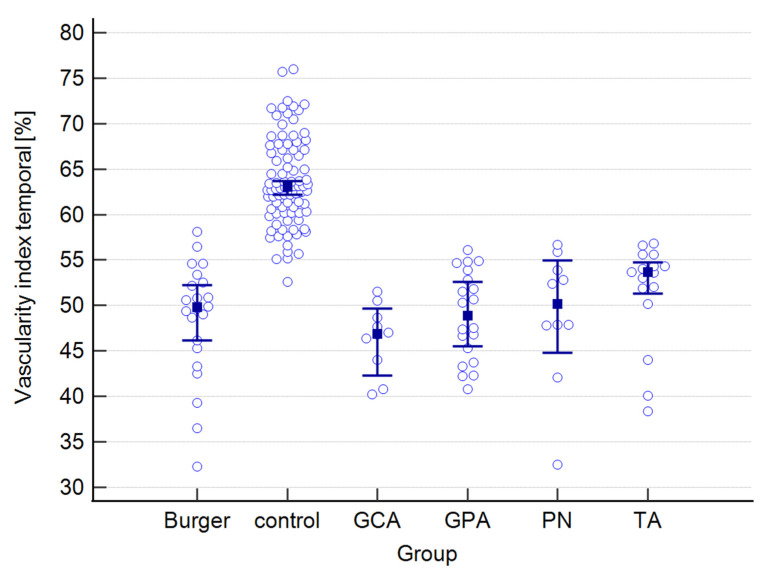
Temporal vascularity index in the study and control groups. Abbreviations: TA—Takayasu’s arteritis, GCA—giant cell arteritis, GPA—granulomatosis with polyangiitis, PN—polyarteritis nodosa.

**Figure 7:**
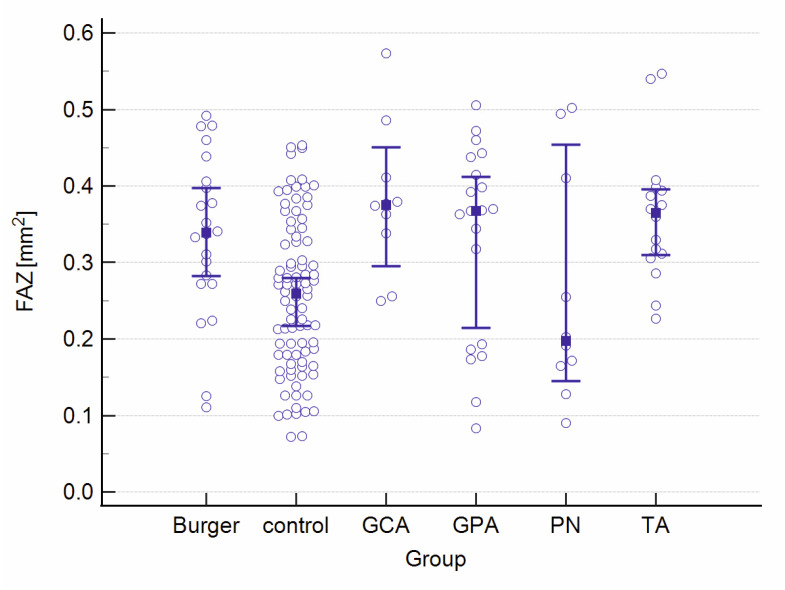
The FAZ area in the study and control groups. Abbreviations: FAZ—foveal avascular zone, TA—Takayasu’s arteritis, GCA—giant cell arteritis, GPA—granulomatosis with polyangiitis, PN—polyarteritis nodosa.

**Figure 8:**
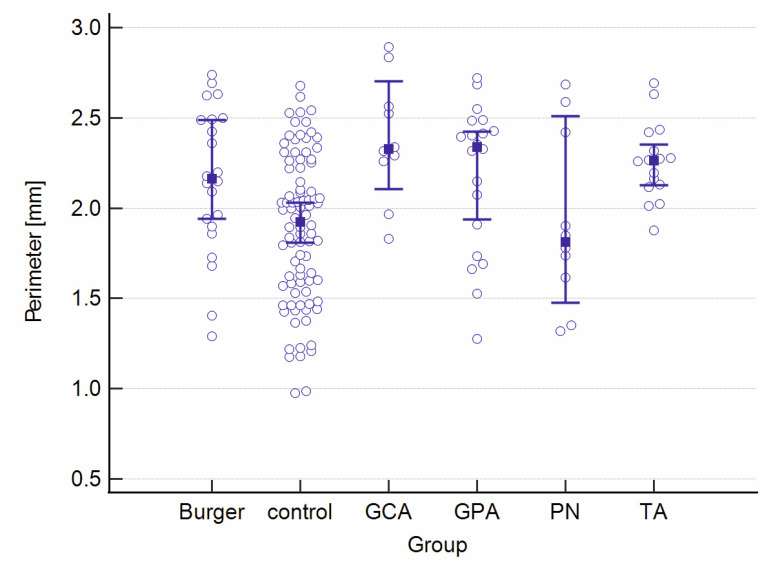
The FAZ perimeter in the study and control groups. Abbreviations: FAZ—foveal avascular zone, TA—Takayasu’s arteritis, GCA—giant cell arteritis, GPA—granulomatosis with polyangiitis, PN—polyarteritis nodosa.

A graphical presentation of the data from Table 1 and Table 2 can be found in Figure 1, Figure 2, Figure 3, Figure 4, Figure 5, Figure 6, Figure 7 and Figure 8. In these figures, the median values of the measured parameters for each disease and the control group are indicated by a blue square, while brackets represent the 95% confidence interval. Subgroups are considered separately in each figure. 

Figure 1, Figure 2 and Figure 3 depict central, nasal, and temporal CTs in both the study and control groups.

The values of central, nasal, and temporal CVIs in the study and control groups are presented in Figure 4, Figure 5 and Figure 6.

Figure 7 and Figure 8 show the FAZ area and perimeter in the study and control groups. 

## 4. Discussion

We have decided to measure two parameters when evaluating the choroid: CT and CVI. The first one is considered to be influenced by many factors–refractive error, gender, and age for example [17,18,19]. That is why a need for a more versatile parameter emerged. In recent years, CVI has become promising in determining the risk of a possible visual impairment. Its decrease has been associated with many ophthalmological conditions (e.g., age-related macular degeneration) as well as systemic diseases with ocular manifestations (e.g., diabetic retinopathy). CVI appears to be less influenced by the same factors as CT, making it a potentially more valuable parameter for choroidal evaluation [20]. For many years, indocyanine green angiography (ICG) has been regarded as an examination dedicated to the assessment of the choroid. Its huge advantage is a much wider field of imaging compared to non-invasive techniques and the ability to characterize the presence of granulomatous lesions in the difficult-to-access peripheral areas [21]. However, its invasive character raises some serious issues as to the safety of the procedure. While OCTA cannot fully replace ICG, especially when diagnosing inflammatory processes, it can be highly beneficial for screening and routine tests and in cases where contrast administration is contraindicated.

The mean central CT value was higher in the study group compared to the control group. While statistical analysis revealed a significant difference, it is worth noting that the results in both groups appear to be within the normal range reported by other authors [22,23,24,25,26]. Higher values of CT in the study group, in comparison to the control group, could be attributed to a subclinical inflammatory process, as proposed by Baytaroğlu et al., in their study on childhood PN [27]. 

Agrawal et al. measured a mean value of CVI in a group of 345 healthy eyes and the result was equal to 65.61 ± 2.33% [16]. In a study on healthy subjects conducted by Xuan et al., the CVI was established to be 69.7% [28]. In all our groups, the values of CVI were lower than the results given by previously mentioned authors and were significantly different from the results of our control group. Decreased CVI values have been documented in diabetic retinopathy [29,30]. Chronic inflammation in vasculitis patients can result in vascular stenosis and subsequent ischemic changes. This observation suggests potential similarities between vasculitis and diabetic retinopathy, possibly accounting for decreased CVI values in both conditions. 

Even though the CT values in our study were similar to the norms in healthy subjects, the CVI values were lower. This shows that, when evaluating the choroid, one should not solely rely on CT but also incorporate CVI measurements. 

The first measured retinal parameter was the area of FAZ. The results of other authors show significant variability when it comes to determining the values of FAZ area among healthy individuals and what is considered a norm. However, the values usually oscillate around 0.2–0.3 [31,32,33,34,35]. Even though the mean FAZ area values in the study and control groups seem to be similar to the previously mentioned norms, a statistically significant difference emerged between these groups, with higher FAZ area values observed in the study group. Information regarding perimeter values in healthy individuals seems scarce. Nevertheless, our findings align with the reported range of 1.7 to 4.3 [35,36,37,38]. As with the FAZ area, the mean FAZ perimeter values were higher in the study group compared to the control group, and these differences were statistically significant. It is important to note that, similarly to the FAZ area, perimeter values may be influenced by individual characteristics such as age [38]. The enlargement of the FAZ area is often described in the course of various retinovascular diseases such as diabetic retinopathy [39,40]. Soules et al. compared healthy individuals with patients suffering from diabetic retinopathy, and the results of FAZ area were, respectively, 0.28 and 0.35 [41]. It is not unlikely that the ischemic systemic changes in patients with vasculitis may cause microcirculatory deficiencies similar to diabetic retinopathy and hence the enlargement of the FAZ area and perimeter in our study group. However, what needs to be remembered is that many factors are believed to have an impact on FAZ area, for example age and gender (though the impact of these factors is debatable) [42,43]. The mean age of our control group was lower than in the study group. The female-to-male ratio also differed (study group—22:19; control group—29:15).

The final assessed parameter was CI. The difference between the study and control groups appeared to be statistically insignificant. This parameter is supposed to provide information about the resemblance of the FAZ shape to a circle. Values closer to 1.0 indicate a more circular FAZ shape [44]. Although this parameter has not been extensively examined so far, we have managed to find mean values in healthy individuals oscillating around 0.7–0.8 [35,37,44,45,46]. Considerably lower values have been described in the course of glaucoma or diabetic retinopathy [45,47]. It seems that vasculitis does not affect CI in the same way as other conditions that compromise perfusion.

The main limitation of the study is the heterogeneity of the patient group. Primary vasculitis, with which all patients were diagnosed, encompasses a broad group of diseases. This heterogeneity is a common challenge when researching rare diseases. All of the studied types of vasculitis are part of the registry of rare diseases published by organizations such as the National Organization for Rare Diseases (NORD), Genetic and Rare Diseases Information Center (GARD) or European Rare Disease Organisation. Gathering a larger sample is extremely difficult, especially in the areas of low prevalence. Scientific data must be obtained on the bases of case reports, case series, and small sample studies. The mean age of our control group was also lower than that of the study group, and the female-to-male ratio was different in both groups. Some parameters are believed to be influenced by age and gender. Unfortunately, the examinations were conducted during the pandemic, making it exceptionally challenging to recruit healthy volunteers, as the fear of contracting COVID-19 deterred individuals from visiting hospitals and outpatient clinics unless absolutely necessary. 

It is also important to look for similar studies conducted on individuals of the same ethnicity, as the results may vary [48]. Unfortunately, we encountered challenges in finding many studies that measure the parameters we examined in a Caucasian population. 

## 5. Conclusions

Vasculitis is a group of diseases affecting various structures of the eyes, including the retina and the choroid. Conducting routine ophthalmological examinations in patients diagnosed with vasculitis with the assessment of the retina and choroid by measuring parameters like CT, CVI, area and perimeter of FAZ, and CI could be beneficial, as it may detect pathological changes before any ocular symptoms alarm the patients. CVI seems to be especially promising for choroidal evaluation, as it appears to be less influenced by various factors compared to CT. However, due to the limited number of ophthalmologically oriented studies on patients with vasculitis, comprehensive analyses are needed for both vasculitis patients as well as healthy individuals in order to establish normative values for many parameters related to retinal and choroidal condition.

## Figures and Tables

**Table 1:** Mean values of choroidal parameters in the study and control groups.

Group	Mean Central Choroidal Thickness (μm)	Mean Nasal Choroidal Thickness (μm)	Mean Temporal Choroidal Thickness (μm)	Mean Central Vascularity Index (%)	Mean Nasal Vascularity Index (%)	Mean Temporal Vascularity Index (%)
Takayasu’s arteritis	355	333	334	52.0	51.5	51.5
Giant cell arteritis	336	319	314	47.4	46.2	46.4
Buerger’s disease	333	323	324	49.1	49.0	48.5
Granulomatosis with polyangiitis	334	327	327	48.7	47.7	48.9
Polyarteritis nodosa	356	347	360	51.2	49.3	49.0
Vasculitis (all types)	341	329	330	49.6	48.8	49.0
Control group	262	368	387	64.5	62.0	63.6

**Table 2:** Mean values of retinal parameters in the study and control groups.

Group	FAZ—Area [mm^2^]	FAZ—Perimeter [mm]	CI
Takayasu’s arteritis	0.36	2.26	0.88
Giant cell arteritis	0.38	2.38	0.85
Buerger’s disease	0.34	2.16	0.89
Granulomatosis with polyangiitis	0.33	2.18	0.83
Polyarteritis nodosa	0.26	1.93	0.82
Vasculitis (all types)	0.34	2.18	0.86
Control group	0.26	1.89	0.87

Abbreviations: FAZ—foveal avascular zone.

## Data Availability

Data sharing not applicable.

## Supplementary Materials

The following supporting information can be downloaded at: https://www.mdpi.com/article/10.3390/jcm12216827/s1, Table S1: Choroidal thickness and vascularity index of patients with vasculitis; Table S2: The area and perimeter of FAZ and circularity index of patients with vasculitis; Table S3: Choroidal thickness and vascularity index of individuals in the control group; Table S4: The area and perimeter of FAZ and circularity index of individuals in the control group.
