# Supplementary material for: Evaluation of Choroidal and Retinal Features in Patients with Primary Vasculitis—An Original Optical Coherence Tomography and Optical Coherence Tomography Angiography Study
Source: J Clin Med. 2023 Oct 29;12(21):6827. doi: 10.3390/jcm12216827 (PMC10648798; doi:10.3390/jcm12216827)
Supplement: Supplementary file 1 [file jcm-12-06827-s001.zip › jcm-2641515-supplementary.pdf]

Table S1. Choroidal thickness and vascularity index of patients with vasculitis.

Patients number	Eye	Central choroidal thickness (μm)	Nasal choroidal thickness (μm)	Temporal choroidal thickness (μm)	Central vascularity index (%)	Nasal vascularity index (%)	Temporal vascularity index (%)
1	OD	363	371	352	59.3	48.3	42.1
1	OS	356	361	347	50.1	47.3	52.8
2	OD	318	320	315	46.2	45.3	46.4
2	OS	289	294	284	45.9	49.8	44.0
3	OD	314	323	316	53.1	51.7	52.5
3	OS	316	324	321	51.6	49.1	50.6
4	OD	327	333	334	54.1	54.9	54.7
4	OS	346	328	335	51.2	49.6	51.8
5	OD	358	345	330	48.8	49.2	48.7
5	OS	362	349	331	47.7	47.2	45.3
6	OD	368	327	348	38.0	32.8	40.2
6	OS	314	297	260	46.1	42.4	40.8
7	OD	333	319	299	53.6	53.6	54.0
7	OS	346	323	311	52.0	52.0	51.9
8	OD	381	357	351	38.2	31.0	38.4
8	OS	360	335	332	56.4	55.4	55.6
9	OD	352	329	318	48.9	51.1	50.5
9	OS	351	331	315	46.3	44.8	47.7
10	OD	330	304	307	54.0	58.0	56.1

10	OS	327	300	305	47.0	49.5	46.8
11	OD	344	315	317	42.4	39.0	43.3
11	OS	359	333	362	46.6	46.4	42.5
12	OD	340	315	342	54.2	51.9	52.8
12	OS	334	307	337	53.9	53.5	54.8
13	OD	318	288	321	53.3	51.3	52.4
13	OS	321	316	338	57.5	56.6	53.9
14	OD	324	314	345	55.2	53.3	55.9
14	OS	328	316	353	56.7	54.4	56.7
15	OD	329	326	346	53.5	51.2	50.2
15	OS	329	338	345	54.0	54.3	55.6
16	OD	375	391	395	33.2	31.9	32.5
16	OS	512	510	557	45.5	47.0	47.8
17	OD	323	325	317	52.0	50.2	50.3
18	OD	332	330	345	38.2	35.2	43.3
19	OD	336	341	328	49.6	49.9	49.4
19	OS	340	345	333	47.1	45.2	49.0
20	OD	330	330	325	54.2	52.6	54.3
20	OS	333	325	326	55.7	54.1	54.3
21	OD	348	341	346	51.0	56.3	51.5
21	OS	323	319	314	52.0	49.1	48.7
22	OD	340	338	332	47.7	47.0	50.8

22	OS	337	335	332	52.7	50.4	54.6
23	OD	358	433	305	45.4	47.6	40.8
23	OS	320	282	299	45.6	41.4	45.3
24	OD	361	331	337	45.6	48.6	39.3
24	OS	337	278	316	46.2	43.3	46.2
25	OD	394	360	367	54.0	53.5	56.6
25	OS	393	356	369	57.2	59.4	56.8
26	OD	372	334	349	50.8	52.6	52.0
26	OS	377	341	352	51.9	48.3	53.6
27	OD	352	318	327	52.4	52.7	53.4
27	OS	318	320	322	49.0	49.8	49.7
28	OD	313	311	302	55.2	58.7	56.5
28	OS	316	310	307	58.5	55.4	58.1
29	OD	324	322	310	53.1	51.9	54.6
29	OS	322	320	308	51.2	52.0	52.2
30	OD	333	336	317	49.6	48.9	51.5
30	OS	336	340	319	47.7	38.2	46.7
31	OD	322	320	311	52.6	50.6	53.9
31	OS	323	324	338	47.1	52.9	47.5
32	OD	314	312	328	52.5	55.6	50.9
32	OS	317	312	332	49.5	52.5	49.9
33	OD	313	313	337	48.8	49.2	47.4

33	OS	312	312	339	43.4	39.0	43.7
34	OD	310	310	332	41.0	40.2	42.2
35	OD	320	294	295	54.0	55.6	53.0
35	OS	329	300	302	55.8	54.9	53.7
36	OD	356	324	330	55.7	55.6	54.9
36	OS	346	311	322	41.8	42.3	42.3
37	OD	375	342	343	46.6	47.6	44.0
37	OS	374	342	339	44.7	48.0	40.1
38	OD	350	318	317	50.1	46.1	46.8
38	OS	351	317	325	49.8	44.5	47.0
39	OD	330	299	298	50.5	51.6	47.9
39	OS	302	308	304	49.3	48.4	44.5
40	OD	384	389	370	49.6	44.5	50.7
41	OD	342	322	344	40.8	36.1	36.5
41	OS	312	296	283	38.5	46.1	32.3

Abbreviations: OD - oculus dexter, OS - oculus sinister

Table S2. The area and perimeter of FAZ and circularity index of patients with vasculitis.

Patients number	Eye	FAZ - area [mm^2]	FAZ - perimeter [mm]	Circularity index
1	OD	0.13	1.32	0.92
1	OS	0.20	1.62	0.98
2	OD	0.41	2.32	0.96
2	OS	0.38	2.89	0.57
3	OD	0.40	2.74	0.67
3	OS	0.46	2.49	0.93
4	OD	0.36	2.15	0.99
4	OS	0.44	2.69	0.76
5	OD	0.35	2.50	0.71
5	OS	0.34	2.14	0.94
6	OD	0.36	2.52	0.72
6	OS	0.37	2.26	0.92
7	OD	0.41	2.32	0.96
7	OS	0.40	2.27	0.97
8	OD	0.55	2.63	0.99
8	OS	0.23	1.88	0.81
9	OD	0.26	1.97	0.83
9	OS	0.25	1.83	0.94
10	OD	0.47	2.55	0.91
10	OS	0.44	2.49	0.90
11	OD	0.34	2.09	0.97
11	OS	0.38	2.36	0.85
12	OD	0.42	2.33	0.96
12	OS	0.40	2.32	0.93
13	OD	0.41	2.42	0.88
13	OS	0.26	1.90	0.89
14	OD	0.49	2.59	0.93
14	OS	0.50	2.69	0.88
15	OD	0.24	2.02	0.75
15	OS	0.29	2.27	0.70
16	OD	0.17	1.78	0.66
16	OS	0.09	1.35	0.62
17	OD	0.12	1.53	0.64
18	OD	0.19	1.91	0.64

19	OD	0.13	1.41	0.80
19	OS	0.11	1.29	0.84
20	OD	0.31	2.16	0.82
20	OS	0.54	2.69	0.94
21	OD	0.49	2.56	0.93
21	OS	0.57	2.83	0.90
22	OD	0.31	2.20	0.81
22	OS	0.30	1.96	0.98
23	OD	0.46	2.49	0.94
23	OS	0.51	2.72	0.86
24	OD	0.37	2.18	0.99
24	OS	0.48	2.63	0.87
25	OD	0.39	2.28	0.95
25	OS	0.32	2.01	0.99
26	OD	0.39	2.26	0.95
26	OS	0.36	2.13	1.00
27	OD	0.28	1.90	0.99
27	OS	0.27	1.86	0.99
28	OD	0.33	2.15	0.91
28	OS	0.49	2.63	0.89
29	OD	0.48	2.69	0.83
29	OS	0.44	2.49	0.89
30	OD	0.32	2.07	0.93
30	OS	0.39	2.41	0.85
31	OD	0.34	2.40	0.75
31	OS	0.37	2.40	0.81
32	OD	0.27	1.94	0.91
32	OS	0.22	1.73	0.94
33	OD	0.37	2.35	0.84
33	OS	0.37	2.43	0.79
34	OD	0.19	1.66	0.88
35	OD	0.37	2.42	0.79
35	OS	0.38	2.44	0.80
36	OD	0.17	1.69	0.76
36	OS	0.18	1.73	0.74
37	OD	0.31	2.12	0.88

37	OS	0.33	2.20	0.86
38	OD	0.34	2.29	0.81
38	OS	0.38	2.34	0.87
39	OD	0.19	1.85	0.70
39	OS	0.17	1.74	0.72
40	OD	0.08	1.28	0.64
41	OD	0.41	2.42	0.87
41	OS	0.22	1.68	0.98

Abbreviations: OD - oculus dexter, OS - oculus sinister, FAZ - foveal avascular zone

Table S3. Choroidal thickness and vascularity index of individuals in the control group.

Patients number	Eye	Central choroidal thickness (μm)	Nasal choroidal thickness (μm)	Temporal choroidal thickness (μm)	Central vascularity index (%)	Nasal vascularity index (%)	Temporal vascularity index (%)
1	OD	254	351	361	61.3	60.3	60.8
1	OS	252	344	354	58.8	57.9	58.3
2	OD	309	386	408	60.6	59.8	60.2
2	OS	309	385	405	58.5	57.7	58.1
3	OD	233	331	339	66.6	63.8	64.5
3	OS	230	290	334	65.0	62.2	62.9
4	OD	260	386	404	68.7	66.2	66.8
4	OS	255	383	403	69.6	65.2	67.6
5	OD	280	401	417	64.9	60.8	63.1
5	OS	274	402	416	65.4	61.5	63.6
6	OD	242	379	402	74.1	69.0	71.8
6	OS	242	378	401	73.1	68.1	70.9

7	OD	271	369	384	65.4	61.7	62.2
7	OS	268	375	390	64.4	60.9	61.4
8	OD	251	343	359	67.2	62.4	63.1
8	OS	250	338	342	60.5	55.9	56.6
9	OD	266	368	382	58.3	55.3	55.7
9	OS	274	370	383	68.6	65.4	65.9
10	OD	249	367	382	68.5	63.8	64.5
10	OS	246	370	379	67.5	64.3	65.0
11	OD	272	383	399	61.5	59.3	59.8
11	OS	278	383	395	57.5	55.4	55.9
12	OD	267	364	388	64.6	62.1	62.6
12	OS	272	371	390	64.1	61.8	62.3
13	OD	254	371	401	65.7	62.8	63.4
13	OS	257	368	400	63.3	60.7	61.3
14	OD	239	350	372	67.9	63.0	63.7
14	OS	245	349	367	64.6	59.7	60.3
15	OD	253	354	376	67.3	63.0	63.5
15	OS	255	359	378	64.9	60.8	61.3
16	OD	223	329	350	73.4	67.0	67.8
16	OS	189	320	339	72.6	66.4	67.1

17	OD	248	370	401	72.1	66.3	67.1
17	OS	252	374	405	73.1	67.2	69.9
18	OD	237	320	341	59.0	56.2	57.5
18	OS	233	317	332	64.1	62.0	62.1
19	OD	268	381	398	71.1	68.5	68.7
19	OS	267	381	401	67.3	64.6	64.8
20	OD	244	348	374	65.8	62.8	63.1
20	OS	248	351	379	65.3	62.4	62.7
21	OD	279	387	398	61.1	60.5	60.6
21	OS	274	383	396	61.3	60.7	60.8
22	OD	280	379	400	58.1	57.4	57.6
22	OS	286	374	401	60.5	59.9	60.1
23	OD	268	384	394	63.6	62.9	63.1
23	OS	266	376	387	58.7	58.0	58.2
24	OD	282	386	404	60.7	60.1	60.2
24	OS	264	390	398	71.7	70.9	71.1
25	OD	238	363	382	62.7	61.7	62.0
25	OS	243	367	385	55.9	54.9	55.1
26	OD	244	360	375	65.9	65.0	65.2
26	OS	255	368	385	63.5	62.6	62.8

27	OD	319	390	402	63.4	62.9	63.0
27	OS	319	393	404	61.6	61.1	61.2
28	OD	241	358	377	64.4	63.6	63.8
28	OS	236	361	379	67.2	66.3	66.5
29	OD	281	381	403	54.5	54.1	55.2
29	OS	282	377	399	57.6	57.1	58.4
30	OD	238	353	380	70.1	69.2	71.7
30	OS	238	355	375	69.8	68.7	71.5
31	OD	244	353	383	64.8	64.0	66.2
31	OS	241	353	381	67.2	66.3	68.7
32	OD	267	369	391	59.2	58.6	60.2
32	OS	277	374	395	51.8	51.3	52.6
33	OD	248	351	369	67.1	66.3	68.6
33	OS	247	348	366	60.6	59.8	62.0
34	OD	247	357	377	65.5	62.2	67.1
34	OS	249	368	387	70.4	67.1	72.1
35	OD	309	383	402	58.6	56.8	59.4
35	OS	313	379	401	57.1	55.8	57.8
36	OD	264	372	392	69.2	66.7	70.5
36	OS	268	381	401	67.7	65.2	69.0

37	OD	277	383	398	61.2	58.5	62.5
37	OS	277	382	396	62.2	59.3	63.6
38	OD	255	366	386	66.4	63.5	67.8
38	OS	259	365	384	66.8	64.0	68.2
39	OD	258	374	393	74.1	70.9	75.7
39	OS	257	374	393	74.4	71.2	76.0
40	OD	284	375	391	57.1	54.8	58.3
40	OS	295	380	398	58.2	56.2	59.3
41	OD	273	365	382	66.7	63.9	68.0
41	OS	271	364	384	62.1	59.5	63.3
42	OD	316	394	412	56.5	54.5	57.6
42	OS	316	391	416	57.8	55.6	58.9
43	OD	252	372	394	71.1	68.1	72.5
43	OS	253	373	393	70.5	67.7	71.9
44	OD	266	381	400	61.6	59.3	62.7
44	OS	262	381	401	62.7	60.6	62.2

Abbreviations: OD - oculus dexter, OS - oculus sinister

Table S4. The area and perimeter of FAZ and circularity index of individuals in the control group.

Patients number	Eye	FAZ - area [mm²]	FAZ - perimeter [mm]	Circularity index
1	OD	0.19	1.67	0.88
1	OS	0.14	1.43	0.84
2	OD	0.13	1.44	0.76
2	OS	0.10	1.22	0.84
3	OD	0.39	2.39	0.86
3	OS	0.39	2.38	0.85
4	OD	0.30	2.06	0.89
4	OS	0.33	2.22	0.83
5	OD	0.27	2.04	0.82
5	OS	0.27	1.94	0.88
6	OD	0.45	2.54	0.88
6	OS	0.44	2.53	0.87
7	OD	0.23	1.86	0.82
7	OS	0.22	1.74	0.90
8	OD	0.38	2.41	0.82
8	OS	0.33	2.31	0.78
9	OD	0.17	1.54	0.90
9	OS	0.18	1.59	0.92
10	OD	0.38	2.36	0.85
10	OS	0.38	2.31	0.91
11	OD	0.18	1.57	0.91
11	OS	0.15	1.46	0.91
12	OD	0.22	1.81	0.83
12	OS	0.20	1.63	0.93
13	OD	0.30	2.09	0.88
13	OS	0.26	1.90	0.92
14	OD	0.28	2.01	0.87
14	OS	0.28	2.03	0.85
15	OD	0.21	1.82	0.81
15	OS	0.20	1.70	0.85
16	OD	0.45	2.68	0.79
16	OS	0.45	2.62	0.83
17	OD	0.40	2.42	0.86
17	OS	0.41	2.48	0.84

18	OD	0.10	1.18	0.92
18	OS	0.17	1.53	0.90
19	OD	0.25	1.86	0.91
19	OS	0.26	1.90	0.89
20	OD	0.30	2.09	0.85
20	OS	0.28	2.03	0.86
21	OD	0.15	1.43	0.94
21	OS	0.18	1.60	0.88
22	OD	0.18	1.60	0.87
22	OS	0.15	1.49	0.87
23	OD	0.23	1.82	0.86
23	OS	0.21	1.74	0.89
24	OD	0.13	1.38	0.84
24	OS	0.27	2.00	0.85
25	OD	0.40	2.40	0.87
25	OS	0.40	2.48	0.82
26	OD	0.35	2.26	0.87
26	OS	0.33	2.22	0.85
27	OD	0.13	1.37	0.85
27	OS	0.11	1.23	0.87
28	OD	0.29	2.07	0.85
28	OS	0.34	2.25	0.85
29	OD	0.07	0.98	0.96
29	OS	0.11	1.21	0.92
30	OD	0.37	2.27	0.90
30	OS	0.35	2.53	0.68
31	OD	0.28	1.99	0.90
31	OS	0.32	2.15	0.89
32	OD	0.16	1.46	0.93
32	OS	0.11	1.18	1.00
33	OD	0.28	2.03	0.84
33	OS	0.28	2.04	0.85
34	OD	0.40	2.34	0.91
34	OS	0.41	2.39	0.90
35	OD	0.10	1.24	0.83
35	OS	0.07	0.99	0.92

36	OD	0.22	1.80	0.85
36	OS	0.22	1.81	0.84
37	OD	0.24	1.91	0.83
37	OS	0.26	2.05	0.76
38	OD	0.30	2.03	0.91
38	OS	0.27	2.05	0.82
39	OD	0.36	2.31	0.84
39	OS	0.37	2.27	0.89
40	OD	0.19	1.64	0.91
40	OS	0.16	1.44	0.98
41	OD	0.26	1.96	0.86
41	OS	0.24	1.84	0.89
42	OD	0.15	1.46	0.87
42	OS	0.16	1.59	0.81
43	OD	0.29	2.10	0.84
43	OS	0.27	2.00	0.85
44	OD	0.19	1.62	0.89

Abbreviations: OD - oculus dexter, OS - oculus sinister, FAZ - foveal avascular zone
